# Engineering Mesenchymal Stem Cells with Nanomaterials for Tumor Microenvironment Regulation and Precision Therapy

**DOI:** 10.1007/s13770-026-00794-5

**Published:** 2026-02-16

**Authors:** Dong-Yong Hong, Jieun Han, Wooram Park

**Affiliations:** 1 Department of Integrative Biotechnology, College of Biotechnology and Bioengineering, Sungkyunkwan University (SKKU), Seobu-ro 2066, Suwon, Gyeonggi 16419 Republic of Korea; 2 Institute for Molecular Metabolism Innovation, Soonchunhyang University, Soonchunhyang-ro 22, Asan, Chungcheongnam 31538 Republic of Korea; 3 Department of MetaBioHealth, Institute for Cross-Disciplinary Studies (ICS), SKKU, Seobu-ro 2066, Suwon, Gyeonggi 16419 Republic of Korea; 4 Institute of Biotechnology and Bioengineering, College of Biotechnology and Bioengineering, SKKU, Seobu-ro 2066, Suwon, Gyeonggi 16419 Republic of Korea

**Keywords:** Mesenchymal stem cells (MSCs), Nanomaterials, Tumor microenvironment (TME), Precision therapy, Drug delivery

## Abstract

**Background:**

The tumor microenvironment (TME) is a major obstacle to effective cancer therapy, driving tumor progression, metastasis, and resistance to conventional treatments. Mesenchymal stem cells (MSCs) have attracted increasing interest as therapeutic vehicles due to their intrinsic tumor-homing capability; however, the therapeutic efficacy of unmodified MSCs remains limited.

**Methods:**

This review examines recent engineering strategies for enhancing MSC therapeutic functionality in TME modulation and precision cancer therapy. Relevant literature was surveyed with focus on nanotechnology-enabled approaches. We analyze key TME features including hypoxia, immunosuppression, and physical barriers, and how various engineering strategies address these challenges.

**Results:**

Engineered MSCs have been successfully transformed into therapeutic “bio-factories” through genetic modification, enabling sustained secretion of cytokines, enzymes, or therapeutic proteins. In parallel, payload-based strategies have established MSCs as effective “Trojan horse” carriers for nanomaterials, chemotherapeutic agents, and oncolytic viruses, allowing precise delivery and active remodeling of the TME. These approaches collectively enhance tumor targeting, therapeutic efficacy, and spatial control within solid tumors.

**Conclusion:**

The integration of MSC biology with nanotechnology provides a powerful platform for regulating the TME and achieving precision oncology. While challenges related to safety, protumorigenic effects, and manufacturing scalability remain, recent advances are rapidly addressing these barriers. Engineered MSC-based therapies hold great promise to revolutionize cancer treatment and overcome the longstanding challenges of solid tumor therapy.

## Introduction

Despite decades of progress in oncology, the effective treatment of solid tumors remains a formidable clinical challenge. While conventional therapies such as chemotherapy and radiation, alongside modern targeted therapies and immunotherapies, have improved patient outcomes, their success is often limited by the complex and adaptive nature of the tumor microenvironment (TME) [1]. The TME represents a highly complex and dynamically evolving niche that is actively orchestrated by malignant cells to facilitate their survival, proliferation, and metastatic progression [2, 3]. It comprises a heterogeneous repertoire of cellular constituents—including cancer-associated fibroblasts (CAFs), endothelial cells, and diverse immune cell subsets—as well as acellular components, most prominently the extracellular matrix (ECM). Collectively, these elements constitute substantial barriers to therapeutic efficacy. The dense, collagen-rich ECM serves as a physical barrier that impedes interstitial transport and limits the bioavailability of therapeutic agents, while sustained hypoxia induces the expression of genes associated with treatment resistance [4]. In addition, an immunosuppressive cellular network—predominantly composed of regulatory T cells (Tregs) and tumor-associated macrophages (TAMs)—actively suppresses both innate and adaptive anti-tumor immunity, thereby compromising the effectiveness of multiple immunotherapeutic modalities [5–7]. Consequently, therapeutic approaches exclusively targeting cancer cells frequently prove insufficient, underscoring the urgent need for innovative strategies aimed at systematically dismantling the pro-tumorigenic architecture of the TME.

In the quest for TME-modulating therapies, mesenchymal stem cells (MSCs) have emerged as a uniquely promising cellular vehicle [8, 9]. MSCs possess a remarkable intrinsic ability to home to tumor sites, a phenomenon driven by their expression of chemokine receptors that recognize ligands highly expressed within the TME [10, 11]. This tumor-homing capability enables MSCs to navigate complex biological environments and localize within both primary tumors and metastatic niches. This inherent tumor-tropism, combined with their low immunogenicity—which permits their use in allogeneic settings—and their potent immunomodulatory functions, establishes them as an ideal platform for delivering therapeutic agents directly into the heart of the tumor [12, 13]. However, the therapeutic impact of native MSCs is often limited, and their function can be subverted by the TME, sometimes leading them to contribute to tumor progression [14]. This dual functional role necessitates sophisticated engineering strategies to enhance their therapeutic efficacy while minimizing pro-tumorigenic risks.

Here, a new paradigm emerges by merging cell biology with nanotechnology. Nano-engineering of MSCs encompasses a spectrum of strategies, including intracellular loading of functionalized nanomaterials, surface conjugation, nanomaterial-assisted gene delivery, and stimulus-responsive hybrid systems, through which MSCs can be transformed into potent, multi-functional therapeutic agents [15, 16]. Nanomaterials can serve as carriers for a diverse array of payloads, from conventional chemotherapeutics and pro-apoptotic proteins to imaging agents and genetic materials [17, 18]. This strategy effectively converts MSCs into “Trojan horses” capable of smuggling a concentrated therapeutic arsenal past the TME’s defenses. Importantly, in this review, nano-engineered MSCs are not viewed simply as delivery vehicles, but as cell-based therapeutic platforms whose function, activation, and efficacy are actively shaped by tumor microenvironmental cues. This synergy between advanced materials science and stem cell biology creates a novel therapeutic paradigm: the use of engineered cells not merely as a passive drug carrier, but as a cell-based therapeutic platforms delivery system that can actively sense and remodel the TME to create a more treatment-permissive state [12, 13, 19]. Distinct from prior reviews that primarily focus on MSC biology or nanomaterial formulation in isolation, this review adopts a TME-centric perspective in which key microenvironmental features act as design drivers for MSC engineering and nanomaterial integration, and systematically integrates both cell-based and cell-free strategies within a unified framework.

This review provides an overview of the rapidly advancing field of nano-engineered MSCs in cancer therapy, with a particular emphasis on their role in modulating the TME. We first outline the defining features of the TME that pose major therapeutic challenges, followed by current strategies for nano-engineering MSCs. We then highlight their key applications in overcoming TME-associated barriers and enabling precision therapy. Finally, we discuss the remaining challenges and future directions for clinical translation of this emerging strategy at the forefront of next-generation cancer treatment.

## The TME as a central target in oncology

### Nanomaterial design principles for MSC-mediated delivery

To strengthen the nanotechnology-focused perspective of this review, it is important to recognize that nanomaterials engineered for MSC-mediated delivery must satisfy design requirements distinct from conventional nanoparticle formulations [20]. Given that MSCs internalize, traffic, and release nanomaterials through distinct cellular pathways, their physicochemical properties must be rationally tuned to ensure intracellular stability, efficient cytosolic trafficking, and controlled activation within the tumor microenvironment. Unlike systemically administered nanocarriers, MSC-shuttled nanomaterials must maintain structural stability within the intracellular environment, prevent premature payload leakage, and respond selectively to tumor-associated biochemical cues [21, 22]. These constraints elevate the importance of engineering parameters such as core–shell architectures, surface chemistry, and degradable linker systems, as well as integrating pH-, enzyme-, redox-, and reactive oxygen species (ROS)-responsive motifs to enable TME-specific activation [23]. Parameters such as particle size, stiffness, degradable linkers, and surface charge critically determine whether nanomaterials preserve MSC viability and migratory behavior while maintaining functional responsiveness to TME-specific cues. Moreover, nanoparticle size, stiffness, and surface charge must be optimized to preserve MSC viability and migratory capacity during loading while simultaneously promoting deep tumor penetration [24]. Recent advances in matrix-degradable polymers, hypoxia-activated nanocarriers, and ROS-labile linkers demonstrate how rational nanomaterial design can synergize with MSC tumor tropism to enhance spatial precision and intratumoral selectivity [25]. Together, these guiding principles form a robust framework for designing next-generation MSC–nanomaterial hybrid systems with improved predictability and translational potential [26]. Expanding the engineering perspective beyond MSC biology, a central goal of MSC-mediated nanotherapy is the development of nanomaterials optimized for safe intracellular loading, stable retention, and stimulus-responsive release.

### Hallmarks of the TME: hypoxia, immunosuppression, and physical barriers

**Fig. 1:**
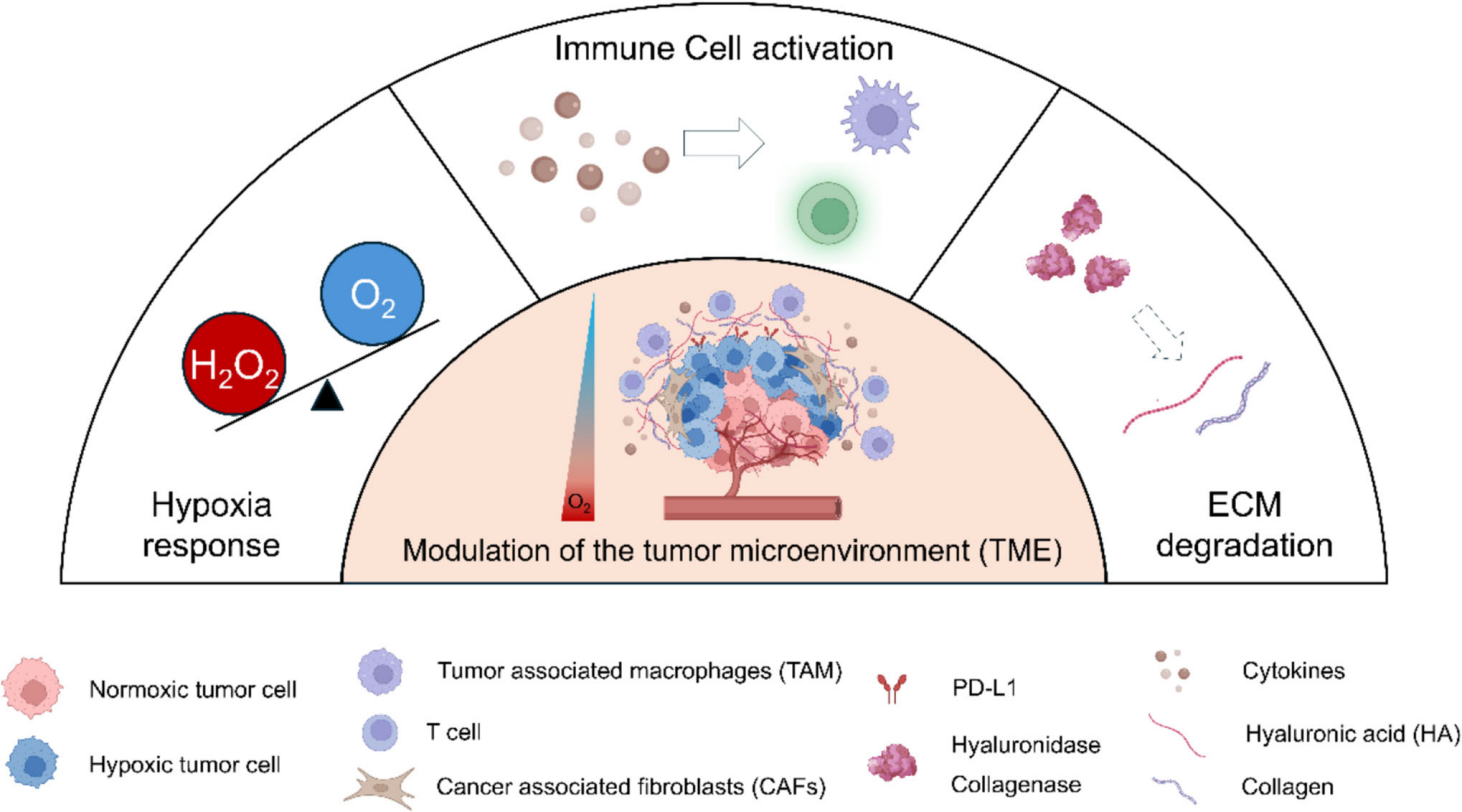
Schematic representation of the tumor microenvironment (TME). The TME is composed of multiple cellular and non-cellular elements that collectively shape tumor progression and therapeutic response. Major cellular constituents include cancer-associated fibroblasts (CAFs), tumor-associated macrophages (TAMs), regulatory T cells (Tregs), myeloid-derived suppressor cells (MDSCs), and endothelial cells, which interact dynamically with tumor cells. The extracellular matrix (ECM) forms a dense fibrotic scaffold that generates solid stress, elevates interstitial fluid pressure, and restricts both immune-cell infiltration and drug penetration. Hypoxia arises from abnormal vasculature and excessive tumor growth, leading to stabilization of hypoxia-inducible factors (HIFs) that drive angiogenesis, metabolic reprogramming, and epithelial–mesenchymal transition (EMT). Immunosuppressive signaling is reinforced through checkpoint pathways such as PD-1/PD-L1 and CTLA-4, contributing to T-cell exhaustion. Collectively, these features define the pathological hallmarks of the TME—hypoxia, immunosuppression, and ECM-derived physical barriers—which jointly foster tumor progression and resistance to therapy. Created with BioRender.com

In the context of this review, these hallmarks are discussed not only as biological obstacles, but as actionable microenvironmental cues that inform the rational design of nano-engineered MSC platforms, as elaborated in subsequent sections. The TME is a complex and dynamic ecosystem that surrounds tumor cells, playing a critical role in cancer initiation, progression, metastasis, and chemoresistance [27]. It is no longer considered a passive bystander but an active participant composed of a diverse array of cellular and non-cellular components, including CAFs, endothelial cells, immune cells, and ECM. This intricate network establishes several key hallmarks that collectively foster a pro-tumoral niche and present significant barriers to effective cancer therapy (Fig. 1).

Hypoxia, or low oxygen tension, is a near-universal feature of solid tumors resulting from rapid cancer cell proliferation outstripping the supply of an aberrant and inefficient tumor vasculature [28–30]. Hypoxia is a major driver of tumor aggressiveness, inducing angiogenesis, epithelial-mesenchymal transition, and metabolic reprogramming through the stabilization of hypoxia-inducible factors (HIFs).

Immunosuppression is another critical hallmark. The TME is often characterized by the infiltration of immunosuppressive cells such as myeloid-derived suppressor cells (MDSCs), Tregs and TAMs [31], which dampen the anti-tumor activity of cytotoxic T lymphocytes (CTLs). The mechanisms that sustain this immunosuppressive state are complex, involving crosstalk between various cell types. For instance, certain stromal cells are known to enhance the suppressive function of Tregs, a mechanism that tumors exploit to evade immune attack [32]. Furthermore, cancer cells can upregulate immune checkpoint ligands like PD-L1, leading to T-cell exhaustion and immune evasion [33, 34].

Finally, the physical barrier formed by a dense and disorganized ECM poses a significant challenge. Overproduction of ECM components like collagen and hyaluronan by CAFs increases solid stress and interstitial fluid pressure, which not only physically impedes the infiltration of immune cells but also limits the penetration and efficacy of therapeutic agents [35, 36].

### Current therapeutic strategies modulating the TME

Recognizing the TME’s pivotal role, several therapeutic strategies have been developed to specifically target its components. Immunotherapies, particularly immune checkpoint inhibitors (ICIs) targeting the PD-1/PD-L1 and CTLA-4 pathways, have revolutionized cancer treatment by reinvigorating the patient’s own immune system to fight cancer [37, 38]. However, their success is often limited to immunologically “hot” tumors with pre-existing T-cell infiltration [39, 40].

Anti-angiogenic therapies aim to “starve” the tumor by inhibiting the formation of new blood vessels, primarily by targeting the vascular endothelial growth factor (VEGF) pathway. While initially promising, the clinical benefit has been modest due to intrinsic and acquired resistance mechanisms, and in some cases, it can even exacerbate hypoxia and metastasis [41, 42].

Stroma-depleting agents, such as enzymes that degrade hyaluronic acid (e.g., PEGPH20), have been explored to break down the dense ECM, thereby improving drug delivery and immune cell access [43]. Yet, the complex and heterogeneous nature of the stroma has made this approach challenging, with mixed clinical outcomes [44].

### Unmet needs and existing callenges in TME-targeted therapies

Despite these advances, targeting the TME remains a formidable challenge. The aforementioned strategies often face limitations such as low response rates, the development of therapeutic resistance, and significant off-target toxicities [45]. The inherent heterogeneity of the TME between patients (inter-tumor) and even within a single tumor (intra-tumor) means that a universal therapeutic strategy is rarely effective. For example, anti-angiogenic therapies can acquire resistance through the activation of alternative signaling pathways, while stroma-depleting agents like PEGPH20 have failed to achieve consistent success in clinical trials due to difficulties in patient selection and the complexity of ECM composition [44]. A critical unmet need is the development of therapeutic platforms that can simultaneously address multiple TME barriers in a targeted and coordinated manner. This necessitates a shift towards more sophisticated, multi-functional therapeutic agents that can not only deliver a cytotoxic payload but also actively remodel the TME to be more susceptible to treatment.

Taken together, these limitations highlight the need for conceptual frameworks that move beyond descriptive characterization of the TME and instead incorporate its biochemical and biomechanical constraints as actionable design inputs for therapeutic development. While many existing approaches primarily regard the TME as a biological obstacle to be overcome, the present review positions the TME as an integrative design reference that informs the rational engineering of nano-engineered MSCs. By emphasizing how TME-associated pressures shape nanomaterial properties, cellular behavior, and therapeutic decision-making, this perspective bridges TME biology with engineering-driven design principles to advance next-generation MSC-based therapeutic platforms.

### Tumor microenvironmental cues guiding nanomaterial engineering for MSC-mediated therapy

Recent evidence highlights that the TME not only governs malignant progression but also serves as a critical design determinant for nanomaterials intended for MSC-mediated delivery. Each physicochemical component of the TME—acidic extracellular pH (6.2–6.8), hypoxic gradients, elevated ROS, dysregulated proteases (e.g., MMP-2/9, cathepsins), and extensive ECM stiffening—directly influences nanomaterial stability, activation, and therapeutic performance [23]. Acidic pH can accelerate the disassembly of pH-responsive lipids or polymers, enabling selective release of therapeutic payloads in tumor regions while preserving particle integrity during MSC trafficking in circulation [46]. Likewise, the dense collagen- and fibronectin-rich ECM restricts cellular motility and nanoparticle penetration, thereby necessitating precise tuning of particle size (typically 30–150 nm), surface charge, and matrix-degradable linkers [47]. Hypoxia-driven HIF-1α signaling enhances the intrinsic tumor tropism of MSCs, but concurrently generates ROS-rich conditions that can be exploited using oxidation-responsive nanomaterials for spatially confined activation [48]. Furthermore, heightened MMP activity within the TME provides enzymatic triggers that can facilitate controlled degradation of peptide-crosslinked nanocarriers, improving intratumoral release efficiency [23]. Integrating these TME-specific biochemical and biomechanical cues into nanomaterial design therefore provides a rational foundation for engineering MSC-based “living” nanomedicines with enhanced selectivity, penetration, and therapeutic precision.

## Engineering MSCs: a versatile platform

**Fig. 2:**
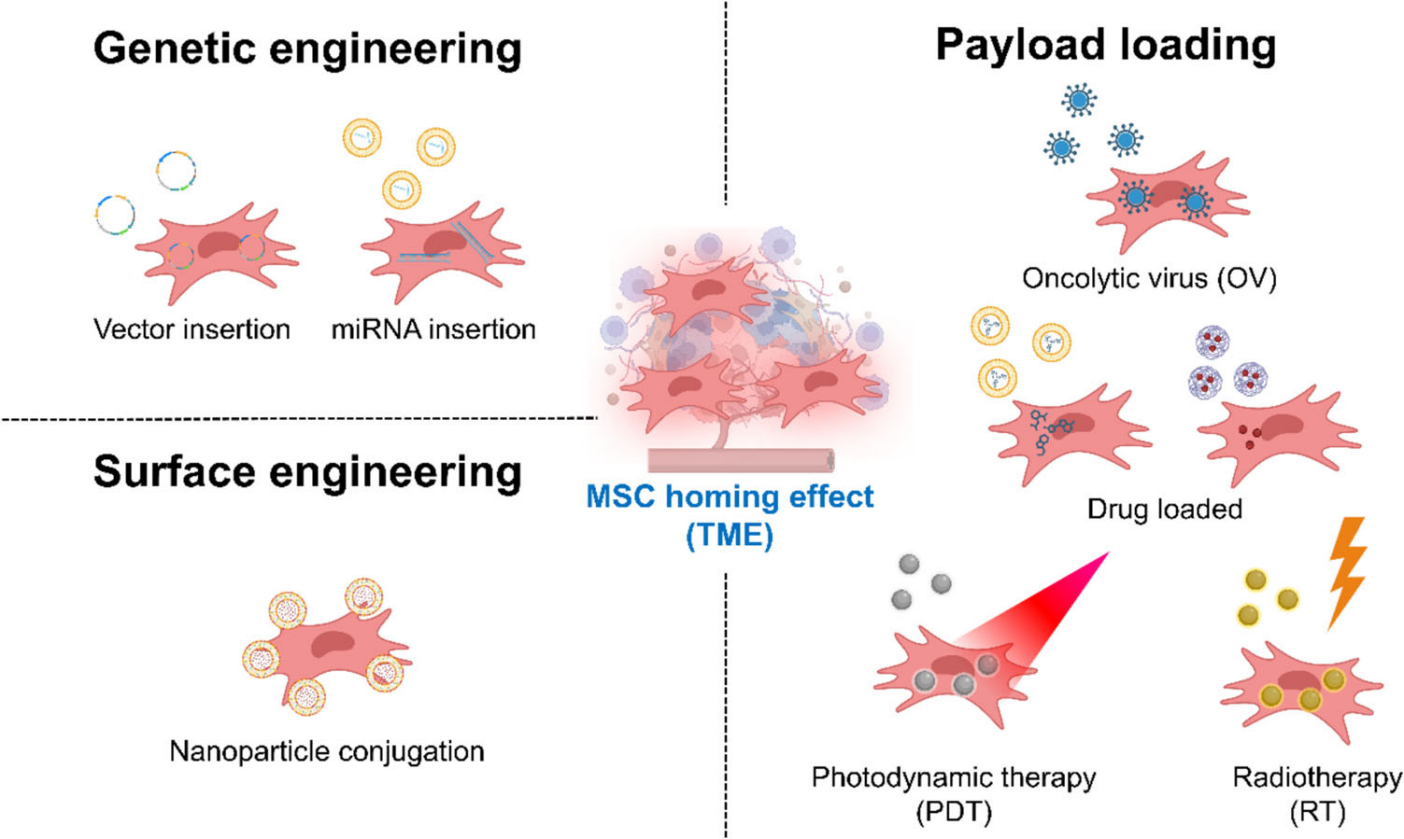
Engineering strategies for mesenchymal stem cells (MSCs). MSCs can be engineered through multiple approaches to enhance their therapeutic efficacy against tumors and to remodel the tumor microenvironment (TME). (i) Genetic modification converts MSCs into “bio-factories” that continuously secrete therapeutic proteins, cytokines, or pro-apoptotic agents following stable or transient gene insertion. (ii) Nanomaterial-based cargo loading leverages the tumor-homing capacity of MSCs to deliver chemotherapeutics, nucleic acids, imaging probes, or oncolytic viruses encapsulated within nanoparticles, either through intracellular uptake or cell surface–anchored nanoparticle conjugation. (iii) Surface engineering functionalizes the MSC membrane with drug-loaded nanoparticles or bio-orthogonal ligands, enabling stimuli-responsive payload release within the TME while preserving intrinsic migratory and immunomodulatory functions. Collectively, these strategies position MSCs as versatile and programmable cellular platforms for precision cancer therapy. Created with BioRender.com

To overcome the challenges presented by the TME, a therapeutic platform is needed that can actively navigate biological barriers, sense the local environment, and deliver a therapeutic payload with high precision. MSCs, endowed with unique biological properties, have emerged as a highly promising candidate for such a “living” drug delivery system. This chapter discusses the rationale for using MSCs and the diverse engineering strategies used to augment their therapeutic capabilities (Fig. 2). Within this framework, different MSC engineering approaches are introduced with an emphasis on their design rationale and application context, providing a comparative perspective for subsequent sections. Collectively, the diverse MSC engineering strategies discussed in this section underscore the versatility of MSCs as programmable therapeutic platforms. These strategies range from genetic modification and payload-based loading to advanced surface functionalization and hybrid approaches. While each strategy offers distinct advantages in terms of mechanism of action and ability to target specific TME barriers, they also present unique limitations related to safety, controllability, and translational feasibility. Table 1 provides a comparative summary of these representative approaches, highlighting their design rationale, primary mechanisms, targeted TME barriers, and key challenges.

**Table 1:** Representative MSC engineering strategies

Engineering strategy	Design / engineering approach	Primary mechanism	Targeted TME barrier(s)	Key findings	Limitations / notes	Representative study
Genetic Engineering	IL-2 secreting MSCs	CD8 + T cell reinvigoration	Cold tumors	Synergy with PD-1 blockade	Potential IL-2 toxicity	[12]
	MSCs expressing TRAIL	TRAIL-induced apoptosis	Apoptosis resistance	Full-length TRAIL more effective	Requires tumor accumulation	[44]
	MSCs expressing IFN-α	IFN-α immune activation	Immune escape	Strong CD8 + activation	Cytokine toxicity	[45]
	MSCs expressing IFN-β	Local IFN-β secretion	Immunosuppressive TME	Tumor inhibition in vivo	Viral vector concerns	[56]
Payload-based	MSCs loaded with SPIO	Magnetic hyperthermia	Poor NP penetration	Improved NP accumulation	MSC migration impact	[50]
	Photoacoustic-triggered NP MSCs	Chemo-photothermal effect	ECM/vascular barrier	Enhanced tumor homing	Light penetration limits	[51]
	MnO2@Ce6 MSCs	O2 generation + PDT	Hypoxia	Enhanced PDT & imaging	ROS stress risk	[56]
	MB + IL-12 MSN-loaded MSCs	ICD + IL-12 immunotherapy	Cold TME	DC maturation & CD8 increase	MSC viability concerns	[60]
	dNP–Ce6 MSCs	Degradable NP release + NIR-II	Poor penetration	Better migration & imaging	NP density affects motility	[65]
Surface Engineering	ROS-responsive NP-tethered MSCs	Stimuli-responsive release	High ROS niches	Improved precision	ROS heterogeneity	[54]
	Liposomeanchored MSCs	Stimuliresponsive release	Inflammation/fibrosis	Lung injury reduction	Homing variability	[55]
Cell-Free EV	PFD + miR138 EVs	CAF suppression + ECM remodeling	Fibrotic ECM	Gemcitabine penetration improved	EV scaling challenges	[63, 64]
	Cell-derived nanovesicles	EV-mimetic delivery	Multi-barrier	High production yield	Lower biological complexity	[76]

TRAIL, TNF-related apoptosis-inducing ligand; SPION, superparamagnetic iron oxide nanoparticles; NP, nanoparticle; PDT, Photodynamic therapy; ICD, immunogenic cell death; ROS, reactive oxygen species; PFD, pirfenidone; CAF, cancer-associated fibroblasts; ECM, extracellular matrix, EV, extracellular vesicles

### The rationale for MSCs in cancer therapy: inherent biological properties

The rationale for selecting MSCs as a cellular vehicle for cancer therapy stems from a unique combination of their innate biological behaviors. Foremost among these is their remarkable tumor-tropic migration. When introduced into circulation, MSCs demonstrate a natural capacity to navigate towards and accumulate within tumor tissues [49–51]. However, this capability can exhibit heterogeneity depending on the MSC source (e.g., bone marrow, adipose tissue, umbilical cord), which is a critical consideration for the design of engineering strategies and final therapeutic efficacy. Beyond their navigational prowess, MSCs are distinguished by their profound immunomodulatory capabilities and low immunogenicity, positioning them as a promising platform for developing “off-the-shelf” cell-based therapies [52, 53]. Importantly, most reported nanoengineering and genetic modification strategies are designed to preserve these intrinsic MSC properties, although subtle variations in viability, secretory activity, or migratory behavior have been observed depending on the specific engineering approach employed.

### Strategies for engineering therapeutic MSCs

The therapeutic efficacy of MSCs can be significantly enhanced through various engineering strategies. These can be broadly categorized into genetic modification, where the cell itself becomes a therapeutic agent, and payload-based engineering, where the cell acts as a targeted delivery vehicle. Each strategy offers distinct advantages in terms of therapeutic control, modularity, and translational flexibility, underscoring the need for a comparative understanding of their respective design principles and application contexts.

#### Genetic engineering: turning MSCs into bio-factories

Genetic modification transforms MSCs into “bio-factories” capable of producing and secreting anti-cancer agents directly within the TME. This is typically achieved through the introduction of a therapeutic gene using either viral or non-viral delivery systems. Viral vectors are widely used due to their high gene transfer efficiency. Lentiviral and retroviral vectors can integrate the therapeutic gene into the host cell’s genome, enabling stable, long-term protein expression. This approach has been successfully used to engineer MSCs to express pro-apoptotic agents like TNF-related apoptosis-inducing ligand (TRAIL), which selectively kill cancer cells [54, 55]. Adenoviral vectors, which do not integrate into the genome, are used for transient but high-level expression of therapeutic proteins such as interferons or interleukins [56]. To mitigate the safety concerns associated with viral vectors, such as immunogenicity and insertional mutagenesis, various non-viral methods have been developed. These include physical methods like electroporation and chemical methods using cationic lipids or polymers to deliver plasmid DNA or mRNA. In this context, nanomaterial-assisted non-viral gene delivery has emerged as a key enabling strategy, in which lipid nanoparticles, polymeric carriers, and inorganic nanomaterials facilitate efficient nucleic acid condensation, intracellular trafficking, and controlled release within MSCs. While traditionally less efficient than viral methods, recent advancements in nanoparticle-based delivery systems have significantly improved the efficacy and safety of non-viral gene delivery to MSCs, making it a clinically attractive alternative [57–59]. Importantly, such nanocarriers allow genetic modification to be achieved with reduced cytotoxic stress, thereby helping to preserve MSC viability and tumor-homing capacity, which are critical for effective *in vivo* performance. Nanomaterial-based gene vectors, including lipid nanoparticles and polymeric carriers, offer enhanced protection of nucleic acids and enable controlled intracellular release, thereby improving MSC transfection efficiency without compromising cellular function. Because MSC viability and homing capacity are particularly sensitive to transfection-induced stress, nanomaterial-assisted gene delivery represents a gentler and more tunable alternative to physical or viral approaches. These advances have expanded the engineering toolbox for MSCs, enabling more tunable and potentially safer genetic modification while largely maintaining key cellular functions relevant to tumor targeting.

#### Payload-based engineering: MSCs as “Trojan Horses”

This approach leverages the inherent tumor-homing ability of MSCs to function as targeted carriers for exogenous therapeutic agents. By loading MSCs with anti-cancer payloads, they can act as “Trojan horses,” navigating through physiological barriers to deliver a concentrated therapeutic dose directly to the tumor site. This strategy aims to enhance local drug efficacy while significantly reducing the systemic toxicity associated with conventional therapies. MSCs can be loaded with various nanomaterials encapsulating a wide range of payloads. These include chemotherapeutics, imaging agents, or genetic materials, which are typically loaded via spontaneous internalization or surface conjugation to the MSC membrane. This strategy leverages the tumor-homing ability of MSCs to increase the local concentration of the therapeutic agent at the tumor site, thereby enhancing efficacy while minimizing systemic toxicity [60, 61]. In a particularly innovative application, MSCs serve as cellular carriers for oncolytic viruses (OVs). OVs are viruses that can selectively replicate in and kill cancer cells. However, when delivered systemically, they are often neutralized by the host’s immune system. By shielding OVs within MSCs, they can act as “Trojan horses” to safely transport the viral payload to the tumor. Once there, the released OVs can infect and lyse cancer cells, triggering not only direct tumor destruction but also a potent anti-tumor immune response [62, 63]. Compared with genetic modification, payload-based strategies provide a highly modular platform for therapeutic delivery, allowing flexibility in cargo selection and dosing without permanent alteration of the MSC genome.

#### Advanced surface engineering and hybrid approaches

Recent advances combine multiple strategies to create more sophisticated engineered MSCs. Cell surface engineering, for instance, involves decorating the MSC membrane with functional moieties. A novel approach involves attaching drug-loaded nanoparticles to the cell surface via bio-orthogonal chemistry. This method not only allows for a high payload capacity without affecting intracellular functions but can also be designed to release the drug in response to tumor-specific stimuli, such as a lower pH or specific enzymes. This creates a “smart” delivery system that maximizes therapeutic efficacy and precision [64, 65]. Beyond payload attachment, surface-engineered nanomaterials can modulate MSC adhesion, membrane receptor clustering, and migratory behavior, highlighting the importance of designing nano–bio interfaces that enhance tumor tropism without perturbing native MSC biology. In this context, hybrid MSC–nanomaterial constructs benefit from synergistic interactions, in which nanomaterials augment targeting or functional responsiveness while MSCs provide biological shielding and extended *in vivo* persistence. These hybrid approaches, which integrate genetic modification with advanced nanomaterial conjugation, represent the next generation of engineered MSC therapies.

## Applications of nano-engineered MSCs for TME-centric cancer therapy

Building upon the engineering platforms described in the preceding section, this section details the application of these modified MSCs to strategically dismantle the pro-tumoral TME. By leveraging their innate tumor-tropism and engineered functionalities, MSCs can serve as living tools to remodel the key pathological features of the tumor, thereby creating an environment more susceptible to anti-cancer treatments. Across these diverse application areas, common design considerations emerge, including the need to balance therapeutic payload loading with preservation of MSC viability and tumor-homing capacity, as well as the importance of context-dependent activation within the TME.

### Re-oxygenating the TME: turning “hypoxic” tumors “responsive”

**Fig. 3:**
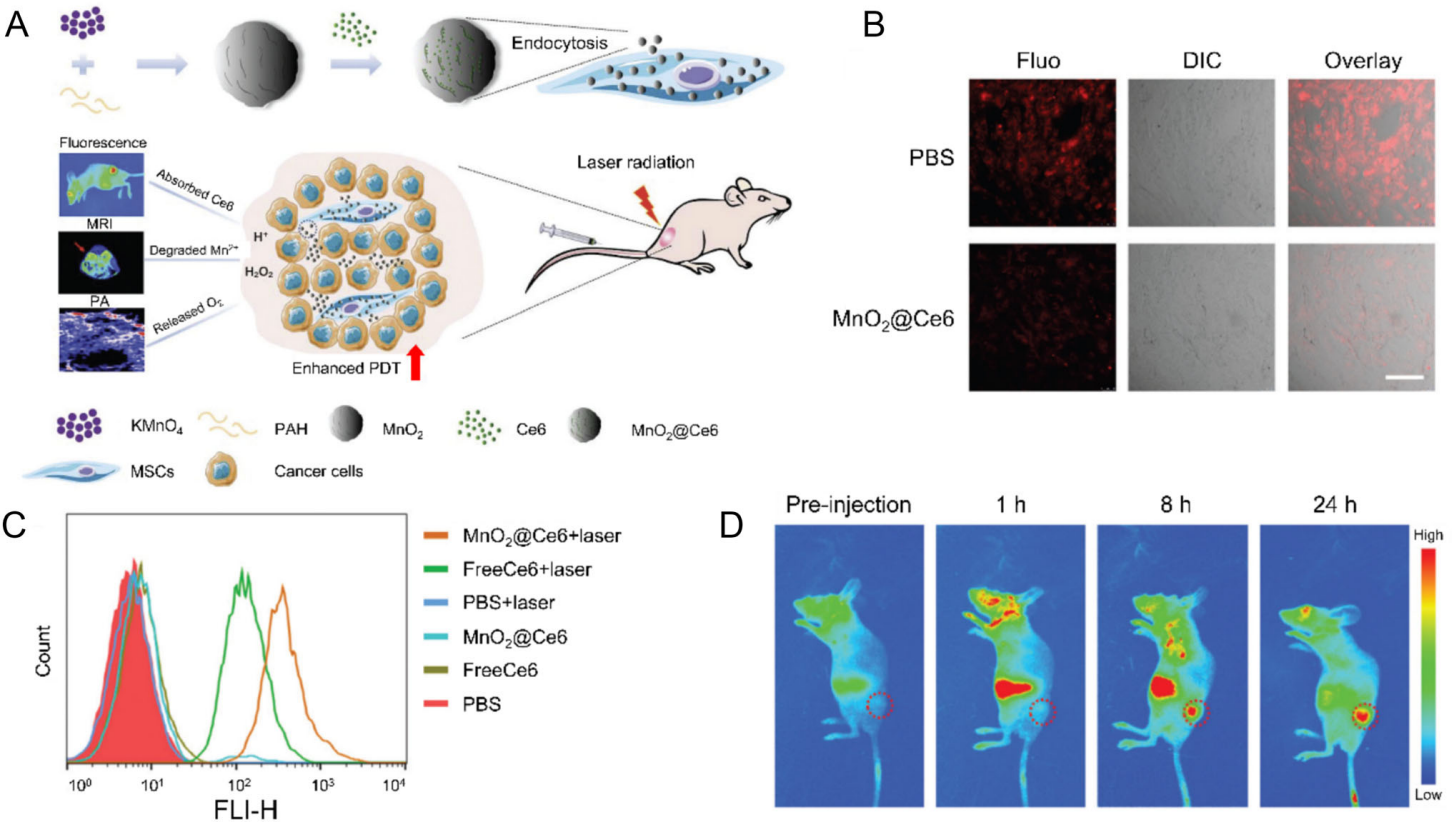
Schematic illustration, intracellular oxygen/reactive oxygen species (ROS) assessment, and multimodal imaging of MnO_2_@Ce6-MSCs. **A** Schematic representation of the preparation of MnO_2_@Ce6-loaded mesenchymal stem cells (MnO_2_@Ce6-MSCs) and their applications in tumor-targeted diagnosis and therapy *in vivo*. **B** Confocal laser scanning microscopy images showing intracellular O_2_ variation in MSCs with or without MnO_2_@Ce6 treatment for 24 h (scale bar: 50 μm). **C** Flow cytometry analysis of ROS generation in MSCs after treatment with PBS, free Ce6, or MnO_2_@Ce6 in the presence or absence of 633 nm laser irradiation. **D** *In vivo* multimodal imaging. Fluorescence imaging of Lewis lung carcinoma (LLC) tumor-bearing nude mice at different time points (1, 8, and 24 h) following intravenous administration of MnO_2_@Ce6-MSCs, demonstrating tumor-specific accumulation and time-dependent biodistribution. Reproduced with permission [66]. Copyright 2020, The Royal Society of Chemistry

To overcome tumor hypoxia, a key driver of malignancy and therapeutic resistance, MSCs can be armed with oxygen-generating nanoparticles. For example, one study loaded MSCs with nanoparticles combining manganese dioxide (MnO_2_) and a photosensitizer (Ce6) (MnO_2_@Ce6-MSCs) (Fig. 3a) [66]. When these MnO_2_@Ce6-MSCs home to the tumor, the MnO_2_ reacts with the high concentration of hydrogen peroxide (H_2_O_2_) within the TME to produce oxygen (O_2_) (Fig. 3b). This localized oxygen generation alleviates hypoxia, enhancing the efficacy of oxygen-dependent therapies like radiotherapy or photodynamic therapy (PDT) (Fig. 3c) [67]. Concurrently, the nanoparticle releases Mn^2+^ ions for T1-weighted magnetic resonance imaging (MRI) and Ce6 provides functionality for fluorescence imaging and PDT, realizing a multimodal imaging-based theranostic platform (Fig. 3d) [66, 68].

### Re-educating the immune microenvironment: turning “cold” tumors “hot”

**Fig. 4:**
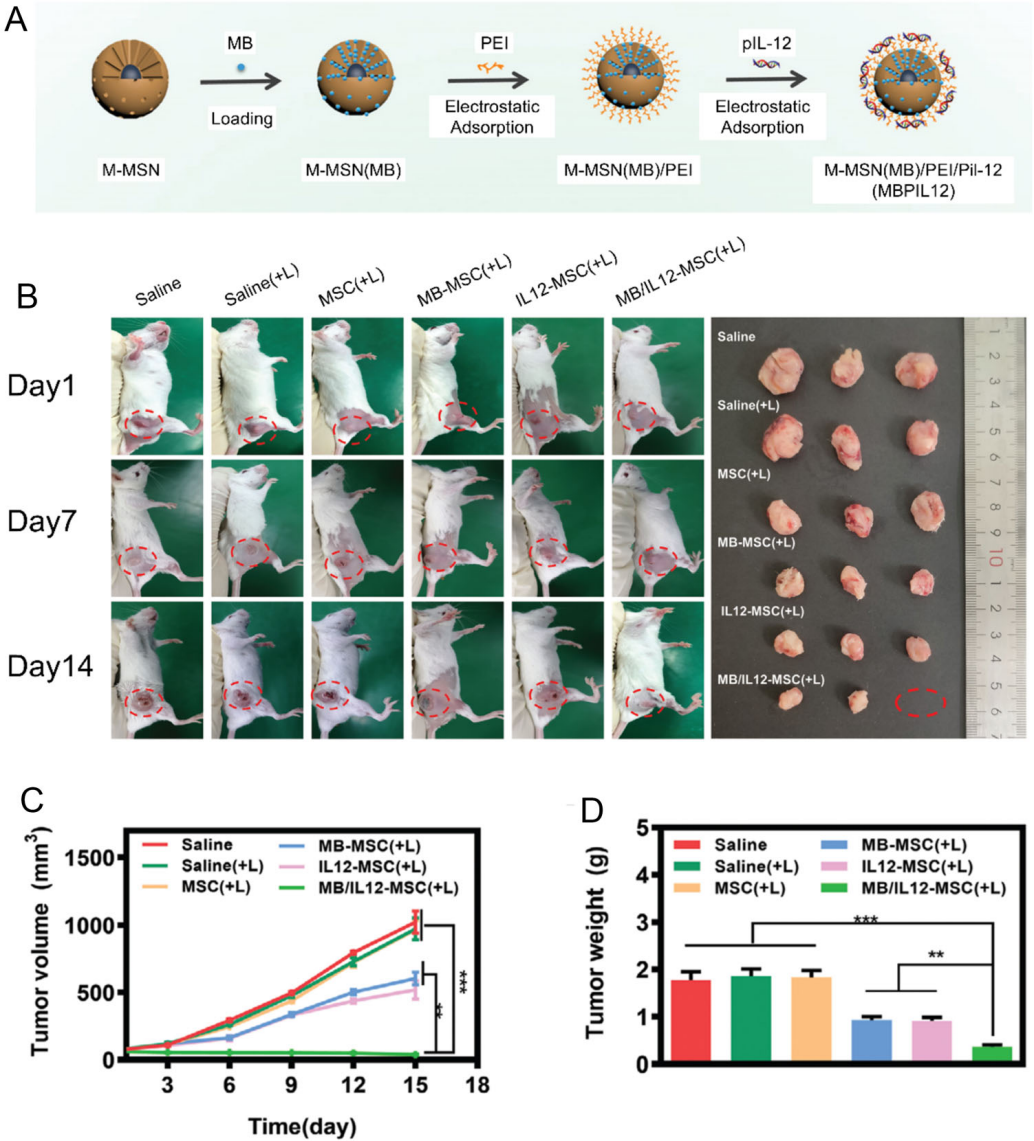
Preparation and *in vivo* anticancer efficacy of MB/IL12-MSCs. **A** Schematic illustration of the preparation of MB/IL12-MSCs: magnetic mesoporous silica nanoparticles (M-MSN) were co-loaded with methylene blue (MB) and IL-12 plasmid (pIL12) to form MBPIL12 nanocomplexes, which were subsequently internalized into MSCs to generate engineered MB/IL12-MSCs. **B** Representative images of tumor-bearing mice at different time points during treatment and photographs of excised tumors from each group at the end of therapy. **C** Tumor growth curves showing significant inhibition of tumor progression in the MB/IL12-MSCs with laser irradiation group compared with control groups. **D** Quantification of tumor weights confirming the most pronounced antitumor effect in the MB/IL12-MSCs group. Reproduced with permission [70]. Copyright 2022, Wiley

Many tumors evade immune destruction by creating an immunosuppressive or “cold” TME. Engineered MSCs can be deployed as immunomodulators to convert these “cold” tumors into “hot” ones that respond effectively to immunotherapy [69]. For instance, one study developed MB/IL12-MSCs by internalizing magnetic mesoporous silica nanoparticles (M-MSNs) co-loaded with an interleukin-12 plasmid (pIL12) and the photosensitizer methylene blue (MB) into MSCs (Fig. 4a) [70]. After these engineered MSCs home to the tumor, an external magnetic field is used to concentrate them at the tumor site, followed by laser irradiation, which elicits two effects. First, MB is activated, inducing immunogenic cell death (ICD) via a photodynamic effect and releasing tumor antigens. Second, pIL12 is simultaneously expressed, leading to the local secretion of the potent anti-tumor cytokine IL-12 (Fig. 4c). This combination therapy promoted dendritic cell maturation and significantly increased the intratumoral infiltration of cytotoxic T lymphocytes, effectively converting “cold” tumors into “hot” ones responsive to immune therapy (Fig. 4b, d) [22, 70, 71].

### Overcoming the physical barrier of the ECM: a cell-free strategy derived from MSCs

**Fig. 5:**
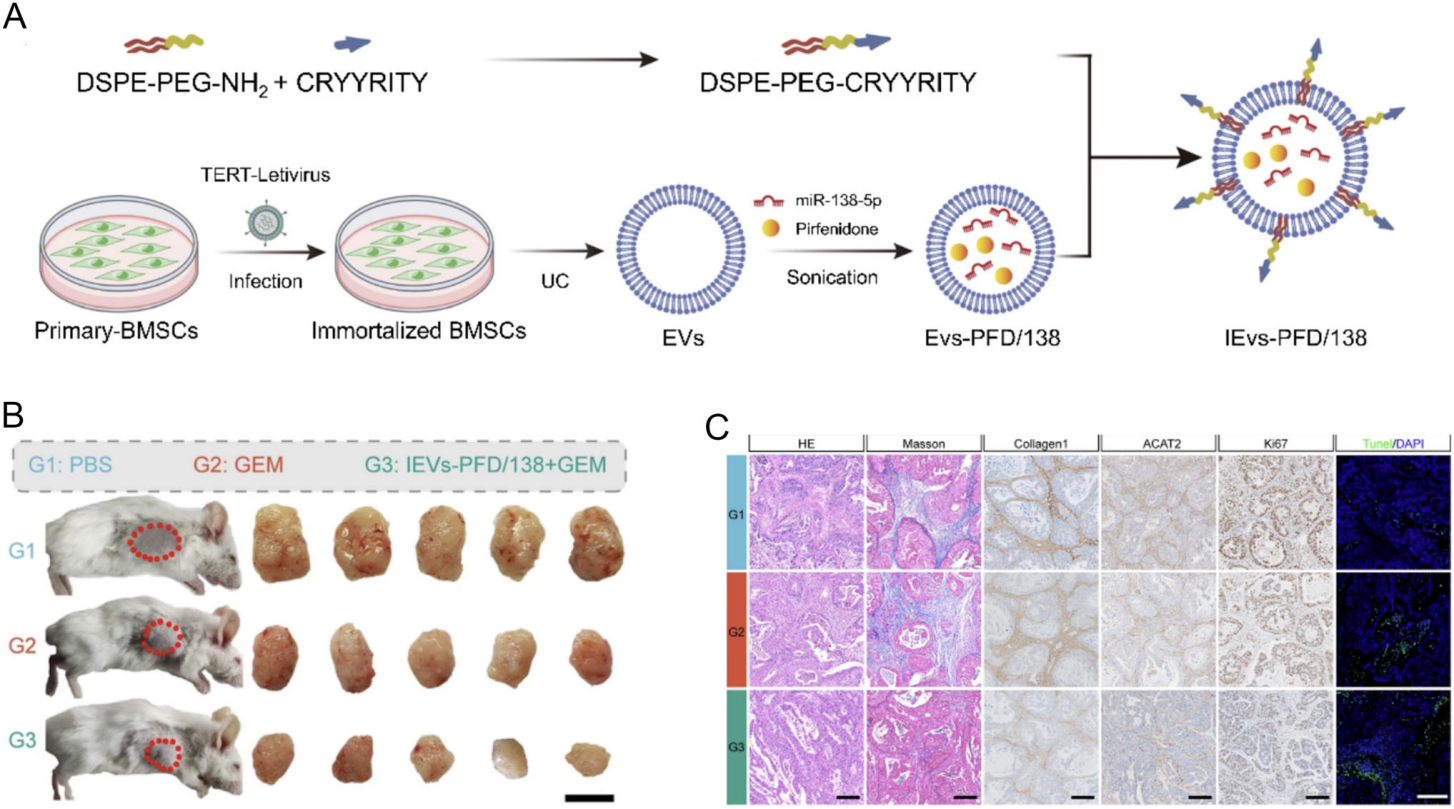
Schematic design and therapeutic evaluation of engineered EVs (IEVs-PFD/138) for pancreatic cancer. **A** Preparation of engineered extracellular vesicles (IEVs-PFD/138). Primary bone marrow MSCs (BMSCs) were immortalized with hTERT lentivirus. EVs were harvested by ultracentrifugation (UC) and subsequently loaded with miR-138-5p and pirfenidone (PFD) via sonication. EV membranes were further modified with DSPE-PEG-CRYYRITY, an integrin α5-targeting peptide, to enhance CAF specificity. **B** *In vivo* therapeutic assessment in a subcutaneous pancreatic cancer mouse model. Representative images of tumor-bearing mice and excised tumors following treatment with PBS (G1), gemcitabine (G2), or the combination of IEVs-PFD/138 and gemcitabine (G3). Scale bar = 1 cm. **C** Histological and immunohistochemical analyses of tumor tissues from each group. Hematoxylin & eosin and Masson’s trichrome staining were performed to evaluate tissue morphology and collagen deposition. Immunohistochemistry of Collagen I, ACTA2, and Ki67 revealed reduced stromal activation and tumor proliferation in the IEVs-PFD/138 + GEM group. TUNEL staining (green) with DAPI counterstaining (blue) demonstrated enhanced apoptosis in the combination-treated group. Scale bar = 100 μm. Reproduced with permission [72]. Copyright 2024, Springer Nature

To breach the dense fibrotic ECM that physically limits immune cell and drug access, a ‘cell-free’ strategy utilizing extracellular vesicles (EVs) derived from MSCs is emerging as a promising alternative to direct MSC injection. In one example, researchers loaded MSC-derived EVs with the anti-fibrotic drug pirfenidone (PFD) and miR-138-5p, which inhibits CAF activity. Furthermore, they decorated the surface of these EVs with an integrin α5-targeting peptide specific to CAFs to create engineered EVs (IEV-PFD/138) (Fig. 5a) [72]. In a pancreatic cancer model, these engineered EVs effectively targeted CAFs, suppressed collagen synthesis, and remodeled the ECM. As a result, the dense stroma was loosened, reducing intratumoral pressure and significantly enhancing the penetration and efficacy of a subsequently administered chemotherapeutic agent (gemcitabine). This approach of engineering MSC secretomes instead of the cells themselves offers a novel possibility to effectively modulate the TME while potentially reducing the safety concerns of cell therapy (Fig. 5b, c) [72, 73].

### Advanced applications in precision delivery and theranostics

**Fig. 6:**
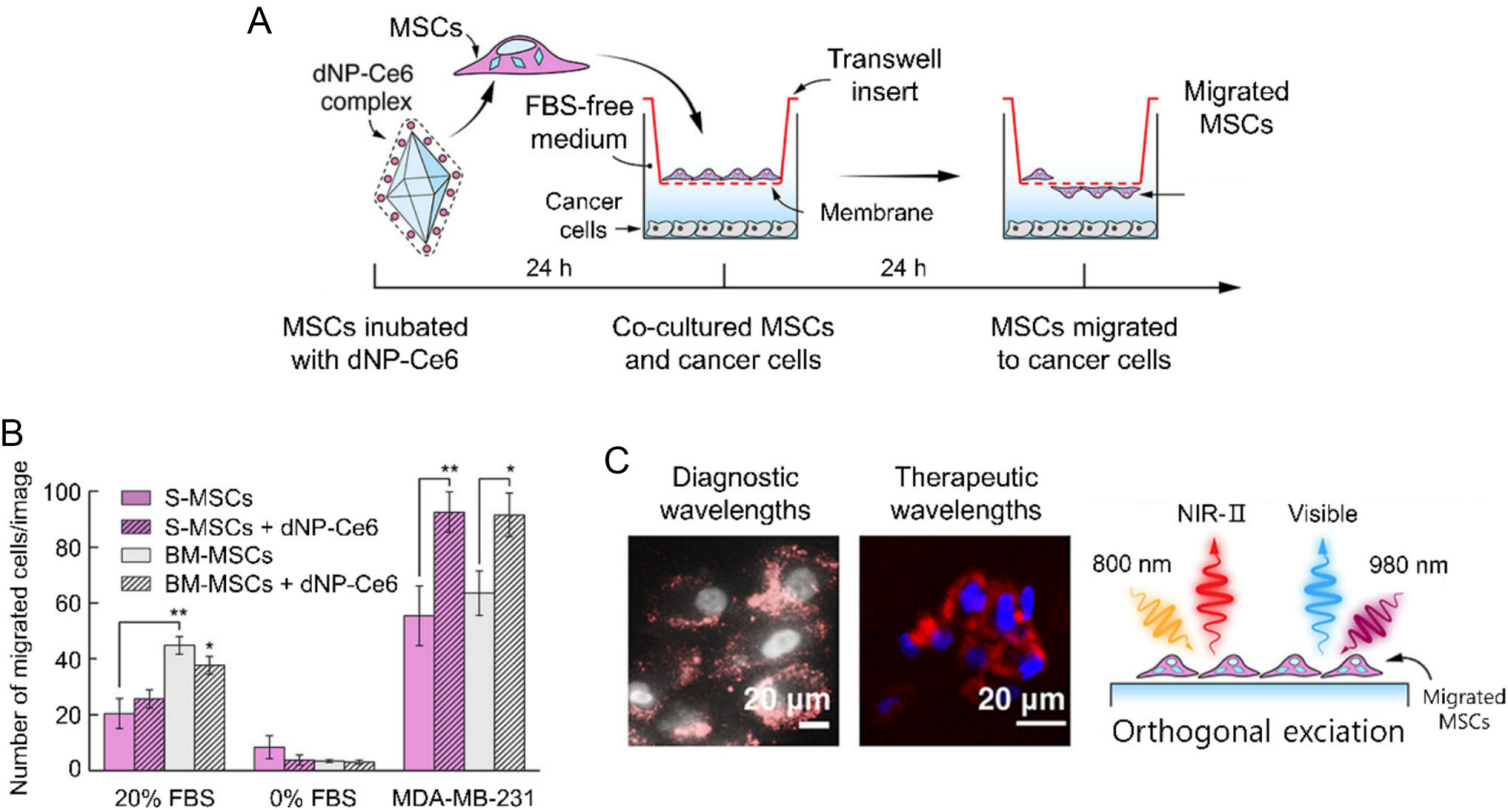
Migration of MSCs loaded with the dNP–Ce6 complex toward cancer cells. **A** Schematic illustration of the Transwell migration assay. MSCs were preincubated with the dNP–Ce6 complex for 24 h, followed by co-culture with MDA-MB-231 breast cancer cells in FBS-free medium using Transwell inserts. After an additional 24 h, the number of MSCs that migrated through the membrane toward cancer cells was evaluated. **B** Quantitative analysis of the number of migrated skin-derived MSCs (S-MSCs) and bone marrow-derived MSCs (BM-MSCs) with or without the dNP–Ce6 complex toward different chemoattractants (20% FBS, 0% FBS, or MDA-MB-231 cells). Both MSC types showed significantly higher migration toward cancer cells compared to FBS, and loading with the dNP–Ce6 complex further enhanced migration (*p ≤ 0.05, **p ≤ 0.01). Error bars represent the standard deviation. **C** Optical imaging of migrated MSCs loaded with the dNP–Ce6 complex. NIR-II luminescence under 808 nm excitation was used for diagnostic imaging (left), while visible upconversion emission under 980 nm excitation confirmed the therapeutic activation potential (right). The orthogonal excitation design allows decoupled diagnostic (NIR-II) and therapeutic (visible PDT) functionalities in migrated MSCs. Scale bars: 20 μm. Reproduced with permission [74]. Copyright 2024, American Chemical Society

For high-precision delivery of anti-cancer agents, the tumor-homing capability of MSCs enables a powerful “Trojan horse” strategy for the highly targeted delivery of various anti-cancer payloads. This approach concentrates the therapy within the tumor, significantly boosting efficacy while minimizing systemic, dose-limiting toxicity. For example, one study loaded MSCs with degradable nanoparticles doped with the photosensitizer Ce6 (dNP-Ce6) (Fig. 6a) [74]. Interestingly, MSCs loaded with these nanoparticles showed enhanced migration capability toward cancer cells compared to unloaded MSCs. Upon irradiation with an external light source after the dNP-Ce6-MSCs migrated to the tumor site, the nanoparticles degraded and released Ce6, exerting a potent photodynamic therapeutic effect on surrounding cancer cells. Moreover, these nanoparticles emitted light in the near-infrared-II (NIR-II) region, enabling real-time tracking of MSC migration. Such MSC-based delivery systems allow for deep tumor penetration of therapeutics, leading to superior anti-cancer effects compared to passive drug delivery (Fig. 6b, c) [66, 74, 75].

#### Theranostics for real-time imaging and treatment

The integration of therapeutic and diagnostic agents onto a single MSC platform has given rise to “theranostic” MSCs. By co-loading MSCs with an imaging agent, such as superparamagnetic iron oxide (SPIO) nanoparticles for MRI, and a therapeutic drug, it becomes possible to non-invasively track their accumulation at the tumor site in real-time. In preclinical glioblastoma models, SPIO-labeled MSCs have been successfully tracked via MRI, confirming their migration towards the tumor [76]. This capability not only validates successful targeting but also allows for the monitoring of therapeutic response, paving the way for personalized treatment strategies.

## Final perspectives and future outlook

The strategies detailed in the previous section highlight the immense potential of nano-engineered MSCs to revolutionize cancer therapy by actively remodeling the TME [77]. However, the path from preclinical success to clinical reality is fraught with significant challenges that must be addressed. This final chapter discusses the key hurdles for clinical translation and explores the exciting future directions that could shape the next generation of MSC-based cancer therapies.

### Key hurdles for clinical translation

The foremost challenge in bringing engineered MSC therapies to patients lies in ensuring their safety and establishing robust manufacturing processes [14]. In this context, the choice of MSC engineering strategy is closely linked not only to therapeutic efficacy but also to regulatory considerations and clinical implementation pathways.

#### Pro-tumorigenic potential and safety assurance of MSCs

While MSCs generally have a good safety profile, they possess a “double-edged sword” characteristic, as they can be subverted by the TME to differentiate into CAF-like cells that promote tumor growth or to enhance their immunosuppressive functions [13, 14]. Key questions remain regarding the long-term fate, biodistribution, off-target accumulation, immunogenicity, and potential tumorigenicity of both the engineered cells and their nanomaterial cargo [78–80]. To address these fundamental concerns, it is essential to validate that the engineering modifications can stably maintain the MSCs in an anti-tumorigenic phenotype and to incorporate ‘safety switches’, such as suicide genes, that allow for the elimination of the infused cells in case of adverse events [81]. Notably, nanoengineering parameters such as material degradability, intracellular persistence, and stimulus-responsive release may critically influence long-term MSC behavior within the TME and should therefore be considered integral components of safety assessment.

#### Manufacturing and quality control

Beyond safety, a critical aspect of therapeutic efficacy is ensuring the long-term engraftment and persistence of the engineered cells. These cell-based therapeutic platforms must survive within the host long enough to exert a sustained therapeutic effect, which requires strategies to manage host immune responses and maintain the stability of the engineered function over time [82]. Concurrently, translating these therapies from the lab to the clinic requires scalable manufacturing processes that comply with Good Manufacturing Practice (GMP) standards [83]. Developing standardized protocols for cell isolation, expansion, engineering, and cryopreservation, along with comprehensive quality control assays, represents a major logistical and technical challenge that is essential for producing consistent, potent, and safe therapeutic products [10]. Accordingly, standardized potency assays that capture both MSC biological function and nanomaterial-mediated therapeutic activity are urgently needed to ensure batch-to-batch consistency.

In this context, a deeper integration of nanomaterial engineering with MSC biology is essential for translating MSC-based nanotherapeutics, as optimized physicochemical properties of nanomaterials directly influence manufacturability, *in vivo* stability, and clinical predictability.

#### Translational challenges & clinical roadmap

Translating nano-engineered MSCs into clinically viable therapeutics requires careful consideration of both therapeutic benefit and biological risk. MSCs can exert pro-tumorigenic effects through immunosuppressive cytokines, angiogenic signaling, and promotion of tumor stemness under certain TME cues [84], highlighting the need for engineering safeguards such as transient gene delivery, tumor-inducible promoters, and suicide-switch systems. Early clinical experience using MSCs as delivery vehicles—most notably in the CELYVIR program, where MSCs transport oncolytic adenovirus—has demonstrated acceptable safety and reproducible tumor tropism in pediatric and adult solid tumors [85], although therapeutic efficacy remains variable. These findings emphasize the importance of standardized manufacturing workflows, potency assays, and rigorous biodistribution monitoring, particularly given the rapid pulmonary sequestration observed following intravenous MSC administration [86] and well-documented inter-donor variability [87]. These translational considerations underscore the need to evaluate how nanomaterial composition, degradation behavior, and release kinetics influence MSC biodistribution and therapeutic persistence *in vivo*. A translational roadmap for nano-engineered MSC therapies should include the selection of tumor indications where TME remodeling offers clear benefit; preference for locoregional delivery to mitigate pulmonary trapping; dosing strategies informed by MSC persistence and payload-release kinetics; and the integration of TME biomarkers—such as immune infiltration patterns and chemokine gradients—to guide patient selection [88]. Together, these elements form a structured foundation for advancing nano-engineered MSCs toward predictable and clinically actionable therapeutic platforms, underscoring that rational nanomaterial and engineering strategy selection is essential for achieving robustness, safety, and reproducibility in clinical settings.

### Future innovations and advanced therapeutic design

Despite the challenges, the field is rapidly advancing towards more sophisticated and effective engineered MSCs. Future iterations will likely be “smart,” multi-functional systems capable of responding to specific TME cues, such as releasing different agents sequentially to first breach the ECM and then attack cancer cells. The true potential of these advanced MSCs may be realized in synergy with other cutting-edge treatments. For example, engineered MSCs could be used to remodel the TME to make it more permissive for CAR-T cell infiltration and activity, or be combined with gene-editing technologies like CRISPR-Cas9 to create even more precise cellular agents.

Beyond direct cell therapy, “cell-free” strategies utilizing engineered cell derivatives such as MSC-derived EVs are emerging as promising alternatives. This approach leverages the therapeutic components of cells while potentially offering improved safety and manufacturability [89–91]. Exploring these cell-free systems alongside whole-cell therapies will broaden the therapeutic landscape. However, designing an optimal nano-engineered MSC—balancing nanoparticle properties, drug loading, and cellular function—remains highly complex. As the design space for nano-engineered MSCs continues to expand, optimizing nanoparticle parameters such as size, surface chemistry, and degradability will increasingly require integrated computational and experimental frameworks to reliably predict therapeutic performance. Artificial intelligence (AI) and machine learning could play pivotal roles in accelerating this process. By analyzing large datasets from preclinical studies, AI algorithms can predict optimal combinations of nanomaterials, drugs, and genetic modifications for specific cancer types and TME characteristics. Moreover, they can optimize complex multivariate parameters, paving the way for truly personalized, cell-based therapies [92]. Investigating these synergistic combinations and leveraging advanced design tools will be key areas of future research, aiming to create multi-pronged attacks against complex diseases like cancer.

## Conclusion

The tumor microenvironment remains a formidable barrier to effective cancer treatment, orchestrating a complex network of physical and biological defenses that shield tumors from therapeutic intervention. This review has delineated an emerging and powerful strategy that leverages nano-engineered MSCs to systematically dismantle these defenses. By capitalizing on the innate tumor-homing ability of MSCs and augmenting their function with a versatile nanotechnology toolbox, it is possible to create sophisticated cell-based therapeutic platforms that can actively remodel the TME.

We have highlighted how these engineered MSCs can counteract key pathological features of the TME, including alleviating hypoxia, re-educating the immunosuppressive milieu, and degrading the dense ECM. Furthermore, their application as high-precision delivery vehicles for a range of anti-cancer agents—from chemotherapeutics to oncolytic viruses—demonstrates their potential to enhance therapeutic efficacy while minimizing systemic toxicity.

While significant hurdles related to clinical translation, particularly in safety and scalable manufacturing, must be overcome, the future of nano-engineered MSCs is exceptionally bright. The progression towards multi-functional, stimuli-responsive systems, their synergy with other advanced therapies like CAR-T, and the integration of AI in their design promise to usher in a new era of personalized and highly effective cancer treatment. Ultimately, the convergence of cell biology and nanotechnology embodied by engineered MSCs offers a transformative approach to cancer therapy, shifting the paradigm from targeting the cancer cell alone to strategically re-engineering the entire tumor ecosystem.

## Data Availability

No datasets were generated or analyzed during the current study.
